# Sonic Hedgehog Promotes Proliferation and Migration of Fibroblast-Like Synoviocytes in Rheumatoid Arthritis via Rho/ROCK Signaling

**DOI:** 10.1155/2022/3423692

**Published:** 2022-06-22

**Authors:** Zaiying Hu, Yingdi Chen, Shangling Zhu, Xiaoxue Feng, Baiyu Zhang, Jianlin Huang

**Affiliations:** Department of Rheumatology, The Sixth Affiliated Hospital, Sun Yat-sen University, Guangzhou, China

## Abstract

**Objective:**

To explore the underlying mechanism of the sonic hedgehog (Shh) signaling pathway in promoting cell proliferation and migration in fibroblast-like synoviocytes (FLS) from patients with rheumatoid arthritis (RA).

**Method:**

FLS were collected from 8 patients with RA and 3 patients with osteoarthritis (OA). The expression of smoothened (Smo, the Shh pathway activator) was quantified by real-time PCR and western blot. FLS were incubated with cyclopamine (a Smo antagonist), purmorphamine (a Smo agonist), Y27632 (a Rho/ROCK signaling inhibitor), or a combination of purmorphamine and Y27632, respectively. Cell proliferation was examined using cell counting kit-8 and cell cycle assays while cell migration was measured with Transwell and wound healing assays.

**Results:**

The expression of Smo was higher in FLS from RA patients than from OA patients (*p* < 0.05). RA-FLS treated with purmorphamine showed significantly activated proliferation (119.69 vs. 100.0) and migration (252.38 vs. 178.57) compared to untreated cells (both *p* < 0.001). RA-FLS incubated with cyclopamine or a combination of purmorphamine and Y27632 exhibited significant suppression of proliferation (81.55 vs. 100.0 and 85.84 vs. 100.0) and migration (100 vs. 178.57 and 109.52 vs. 185) ability (all *p* < 0.001).

**Conclusion:**

Our results demonstrated that Shh promoted cell growth and migration of FLS in RA patients through the Rho/ROCK signaling pathway.

## 1. Introduction

Synovial hyperplasia in patients with rheumatoid arthritis (RA) has always resulted in cartilage and bone degradation [1, 2]. As a major component of the synovial pannus, fibroblast-like synoviocytes (FLS) act as passive responders and imprinted aggressors to elicit destructive joint inflammation [3, 4]. RA-FLS are considered hyperplastic, invasive cells exhibiting multipotent inflammatory properties. However, how RA-FLS acquire and sustain such aggressive phenotypes remains unknown.

The Rho GTPase family is a division of small (~21 kDa) monomeric G proteins that belong to the Ras superfamily of GTPase. Rho GTPases play a key role in cell motility and polarity, axon guidance, vesicle trafficking, and the cell cycle by regulating cytoskeletal polymerization [5]. There are 20 Rho GTPase genes identified in humans; three of them, Rho, Rac, and Cdc42, have been investigated most extensively in recent decades [6]. ROCK is a key kinase in this signaling pathway. Some evidence has shown that the Rho/ROCK signaling pathway is able to regulate proliferation and migration of RA-FLS [7]. However, the underlying mechanisms regulating the effects of Rho/ROCK on RA-FLS have not yet been elucidated.

The hedgehog (Hh) pathway is important for early embryonic development. Hh provides cells with positional information and fate instruction during embryogenesis. After development, Hh mediates tissue homeostasis and wound healing [8, 9]. A growing body of evidence indicates that the aberrant activation of Shh, one of the three subgroups of Hh, is involved in the pathogenesis of a wide range of human diseases, including various types of cancer [9] and autoimmune diseases [10–12]. Smoothened (Smo) is a seven-pass transmembrane protein that acts as a potential Shh signaling pathway activator [13]. Smo inhibitors, including vismodegib, sonidegib, and XL-139, have been used as targeted therapy in basal cell carcinoma and many other tumors in clinical trials [14].

In the noncanonical Shh signaling pathway, Smo stimulates the activation of Rho GTPase [15]. In previous studies, we demonstrated that Shh signaling was overactivated in RA-FLS [11, 16, 17]. We revealed that the proliferation and migration of RA-FLS were remarkably enhanced by a Smo agonist (purmorphamine) and significantly suppressed by a Smo antagonist (cyclopamine), indicating that Shh signaling contributes to abnormal tumor-like behaviors, such as the growth and aggressiveness of RA-FLS [18]. In addition, we observed that Smo activated key molecules in Rho GTPase signaling, such as RhoA and Rac [19]. However, it remains unknown whether Shh signaling promotes RA-FLS proliferation *via* activating Rho/ROCK. Therefore, in this study, we aimed to explore the underlying mechanism(s) of the Shh signaling pathway in promoting growth and investigate the crosstalk between Shh signaling and RhoA/ROCK signaling in RA-FLS.

## 2. Materials and Methods

### 2.1. Patients and Samples

Fibroblast-like synoviocytes were collected from RA and osteoarthritis (OA) patients. The RA patients were diagnosed and identified according to the classification criteria defined by the American College of Rheumatology and revised in 1987 [20]. The 28-joint disease activity score calculated using erythrocyte sedimentation rate (DAS28-ESR) was applied as a disease activity index of RA. The patients exhibited moderate to severe disease activity (DAS28-ESR > 3.2). To collect synovial tissues during knee arthroscopy and replacement, one male and seven female Han Chinese RA patients with an average age of 51.5 ± 8.02 years and one male and two female Han Chinese OA patients with an average of 58.67 ± 5.51 (control group) were recruited from the Third Affiliated Hospital of the Sun Yat-sen University in Guangzhou, China, from July 2016 to December 2019. With a long disease course (≥10 years), most of them had irregular use of disease-modifying antirheumatic drugs (DMARDs). The research was approved by the Medical Ethics Committee of the hospital, and all patients provided signed informed consent.

### 2.2. Propagation and Phenotyping of Primary FLS

To prepare RA-FLS, spliced RA synovial tissues were resuspended in Dulbecco's modified Eagle's medium (DMEM) (Hyclone Laboratories, Losan, UT, USA) containing 10% fetal bovine serum (FBS) (Gibco Laboratories) and transferred to tissue culture flasks. Within the first two weeks, primary FLS began to migrate out of the biopsy tissue. After the FLS expanded to approximately 80% confluency, the formed monolayers were trypsinized and resuspended for propagation. Seeded FLS were morphologically identified by observation with light microscopy. Surface markers, CD14, CD55, CD68, and CD90 (all conjugated antibodies came from Biolegend, USA), in the third or fourth generation of cells were stained, evaluated, and analyzed with a fluorescence-activated cell sorting (FACS) Calibur flow cytometer system (Becton Dickinson, Franklin Lakes, NJ, USA). The third- to fifth-generation FLS were generally used for experiments.

### 2.3. RNA Isolation and Real-Time PCR Analysis

Total RNA was isolated using TRIzol reagent (Invitrogen Life Technologies, Santa Clara, CA, USA), and cDNAs were synthesized using the Prime Script RT Reagent kit (Takara Biotechnology, Dalian, China) according to the manufacturer's instructions. Quantification of the expression of human Smo and glyceraldehyde 3-phosphate dehydrogenase (GAPDH) mRNAs was performed using the SYBR Premix Ex TaqTM kit (Takara Biotechnology) on an ABI-7500 Thermal Cycler (Applied Biosystems Inc., Foster City, CA, USA) according to the manufacturer's instructions. All experiments were examined in triplicate, and positive and negative controls were included. The relative levels of mRNA in FLS from RA and OA patients were quantified using the comparative delta Ct method. The primers used for amplification were as follows (forward, reverse): Smo: forward: 5′-CCT GCT CAC CTG GTC ACT C-3′, reverse: 5′-CAC GGT ATC GGT AGT TCT TGT AG-3′ and GAPDH: (5′-GGA TAT TGT TGC CAT CAT TT T-3′, 5′-AAT GAT GGC AAC AAT ATC CT dT-3′).

### 2.4. Western Blot

A total of 30 *μ*g of protein extracted using RIPA buffer (Cell Signaling Technology, Beverly, MA, USA) was subjected to 10% sodium dodecyl sulfate-polyacrylamide gel electrophoresis (SDS-PAGE). The transferred and blocked polyvinylidene fluoride (PVDF) membrane was then incubated overnight at 4°C with primary antibodies against the rabbit anti-SMO (1 : 1000, Affinity Biosciences, OH, USA), phosphorylated myosin phosphatase targeting subunit 1 (p-MYPT1, 1 : 1000, Cell Signaling Technology, Beverly, MA, USA), MYPT1 antibody (1 : 1000, Cell signaling Technology, Beverly, MA, USA), and RhoA Rabbit mAb (1 : 1000, Cell signaling Technology, Beverly, MA, USA). Subsequently, the membranes were probed with horseradish peroxidase-conjugated secondary antibodies. The immobilized bands detected with the enhanced chemiluminescent (ECL) system were semi-quantified using Alpha View software (San Jose, CA, USA). The expression of *β*-actin or GAPDH was used as an internal standard.

### 2.5. FLS Proliferation Assay

Cell proliferation rates were determined with a cell counting kit-8 (CCK8) assay (Dojindo, Tokyo, Japan) following the manufacturer's instructions. FLS at a density of 2.5 × 10^4^ mL^−1^ were seeded into 96-well plates for 12 hours, followed by individual treatment with 10 *μ*M cyclopamine (Selleckchem, Houston, TX, USA), 1 *μ*M purmorphamine (Sigma-Aldrich, St. Louis, MO, USA), 20 *μ*M Y27632 (Selleckchem, Houston, TX, USA), or a combination of 20 *μ*M Y27632 and 1 *μ*M purmorphamine. Specifically, cyclopamine or purmorphamine dissolved at 10 mM in dimethyl sulfoxide (DMSO) was diluted to the final concentration using DMEM containing 10% FBS. Cells in the control group were treated with vehicle only.

### 2.6. FLS Cell Cycle Assay

For the cell cycle assay, FLS at a density of 4 × 10^5^ mL^−1^ were seeded into six-well plates for 24 h, followed by individual treatment with 20 *μ*M cyclopamine, 1 *μ*M purmorphamine, or a combination of Y27632 and purmorphamine for 48 h. After treatment, the FLS were collected and washed with phosphate-buffered saline (PBS) and transferred into 70% ethanol on ice for 2 h. Afterward, 10 *μ*g/mL propidium lodide (Biotechnology, Shanghai, China) was added and incubated with the cells for 30 min in a dark room at room temperature. Finally, the cell cycle was evaluated and analyzed with a FACSCalibur flow cytometer system.

### 2.7. FLS Migration Assay

Given that the Transwell assay and wound healing experiments are the recognized methods for assessing cell migration [21–23], we used these methods to examine the biological features of the FLS. The migration capacities of the FLS were determined using 8 *μ*m pore Transwell chambers (BD Biosciences, Heidelberg, Germany) in 24-well plates. For the migration assay, FLS at a density of 4 × 10^5^ mL^−1^ were seeded into six-well plates, followed by individual treatment with 20 *μ*M cyclopamine, 1 *μ*M purmorphamine, 10 *μ*M Y27632, or a combination of Y27632 and purmorphamine for 48 h. Then, 100 *μ*L of trypsinized FLS resuspended in serum-free medium was loaded into the upper chamber of the Transwell insert at a density of 8 × 10^4^ mL^−1^. Meanwhile, 600 *μ*L of medium containing 10% FBS was added to the lower chamber. After 12 h incubation at 37°C, the migrated FLS on the bottom insert were fixed with 4% paraformaldehyde and the migration capability was qualified under an inverted microscope by cell counting in five random fields for each membrane at 100x magnification.

The migration activity of the FLS was tested with a wound healing experiment at the same time. Briefly, FLS at a density of 4 × 10^5^ mL^−1^ were seeded into six-well plates for 48 h. To reduce the cell proliferation, 10 *μ*g/mL Mitomycin C (Sigma-Aldrich, St. Louis, MO, USA) was added to the cells for 2 h. After washing with PBS, a straight scratch was made in the wells using a 200 *μ*L pipette tip, followed by individual treatment with 20 *μ*M cyclopamine, 1 *μ*M purmorphamine, or a combination of Y27632 and purmorphamine for 24 h. The scratch width was tested and analyzed with ImageJ at 0, 12, and 24 h.

### 2.8. Statistical Analysis

Statistical analysis was performed using SPSS version 20.0 (Chicago, IL, USA). The measured values were presented as means ± standard deviation (S.D.), or median values and interquartile ranges (IQR) based on at least triplicates. The normality of data was examined by Shapiro-Wilk test and homogeneity of variances was examined by Levene test. Comparisons in two groups were performed using independent sample Student's *t*-test. Statistical differences among groups were tested by one-way analysis of variance (ANOVA). Post hoc comparisons were made by Dunnett's test. *p* < 0.05 was considered to indicate a statistically significant difference.

## 3. Results

### 3.1. Phenotyping of FLS

**Figure 1:**
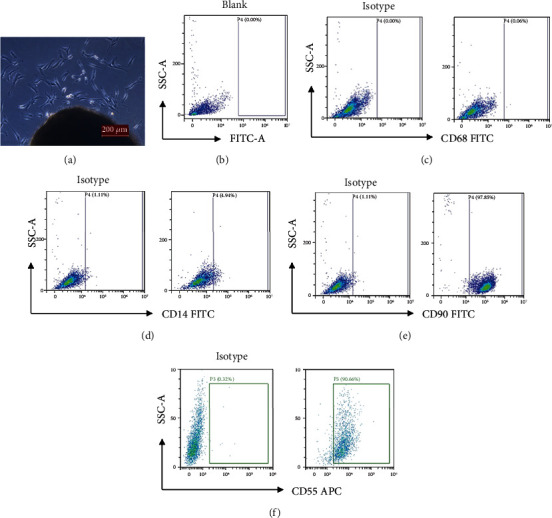
The phenotyping of FLS. (a) FLS began to migrate out of the biopsy tissue after 14 days of incubation. (b–f) Flow cytometry analysis of FLS. (b) Blank staining of FLS. (c) The staining for CD68 was negative. (d) The staining for CD14 was negative. (e) The positive rates for CD90 were over 95%. (f) The positive rates for CD55 were over 90%.

As shown in Figure 1(a), FLS migrated out of the biopsy tissue after 14 days of incubation. FLS from the third to fifth generation were used for the subsequent experiments. Flow cytometry analysis showed that the positive rates for CD90 on FLS were over 95% (Figure 1(e)) and the positive rates for CD55 were over 90% (Figure 1(f)). However, the tests for CD68 (Figure 1(c)) and CD14 (Figure 1(d)) were negative. These results were consistent with previous reports stating that RA-FLS have a high level of CD90 and CD55 expressions and a low level of macrophage markers such as CD68 and CD14 [24, 25], suggesting the FLS we isolated and cultured have the typical phenotype for RA-FLS.

### 3.2. Smo Expression Was Higher in RA Patients than in OA Patients

**Figure 2:**
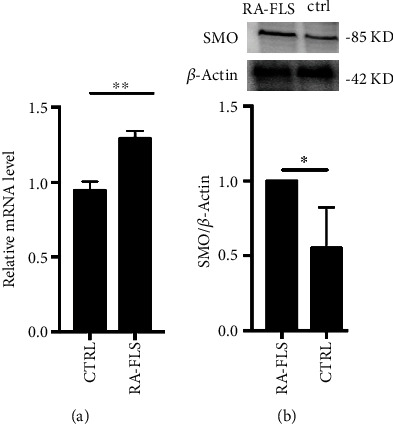
Smo expression was higher in RA patients than in OA patients. (a) The Smo mRNA level in FLS from RA patients was higher than that in FLS from OA patients (^∗∗^*p* < 0.01). (b) Western blot assays demonstrated that the expression of Smo in RA-FLS was also higher than that in OA-FLS (^∗^*p* < 0.05).

The relative mRNA level and the western blot assay both demonstrated that Smo was over expressed in RA patients. As presented in Figure 2(a), the Smo mRNA level in FLS from RA patients was higher than that in FLS from OA patients (1.35 vs. 0.96, *p* < 0.01). As shown in Figure 2(b), the western blot assay demonstrated that the expression of Smo in RA-FLS was also higher than that in OA-FLS (1.03 vs. 0.65, *p* < 0.05).

### 3.3. Smo Agonist and Antagonist Regulate the Expression of Rho/ROCK-Related Proteins

**Figure 3:**
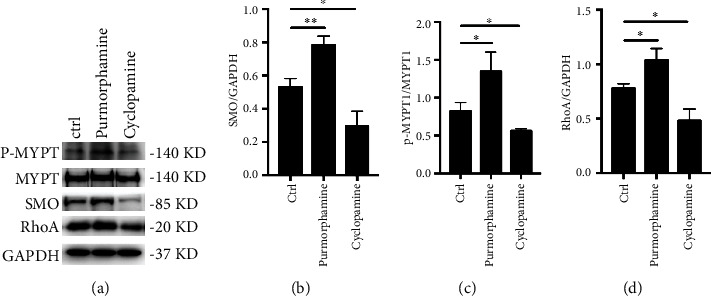
Smo agonist and antagonist regulate the expression of Rho/ROCK-related proteins. (a) The expression of p-MTPT1, MTPT, RhoA, and Smo was detected by western blot. (b) The relative expression of Smo was upregulated in the treatment of purmorphamine, and it was downregulated by using cyclopamine. (c) The phosphorylation level of MYPT-1The was upregulated in the treatment of purmorphamine, and it was downregulated after adding cyclopamine. (d) The relative expression of RhoA was upregulated in the treatment of purmorphamine, and it was downregulated by using cyclopamine. ^∗^*p* < 0.05, ^∗∗^*p* < 0.01.

To assess the activation of the Rho/ROCK signaling pathway, we measured the expression of RhoA and Smo protein, as well as the phosphorylation level of MYPT-1 in RA-FLS treated with purmorphamine or cyclopamine. As shown in Figure 3, western blot analysis showed that the expression levels of p-MYPT1 and RhoA were significantly upregulated in the purmorphamine treatment group compared to the levels in the control group (1.34 vs. 0.82 and 1.04 vs. 0.78, both *p* < 0.05). In contrast, the expression of p-MYPT1, RhoA, and Smo was downregulated significantly by blocking Smo using cyclopamine (0.56 vs. 0.82, 0.48 vs. 0.78 and 0.29 vs. 0.53, respectively, all *p* < 0.05).

### 3.4. Shh Activation Induces RA-FLS Proliferation *via* Rho/ROCK Pathway

**Figure 4:**
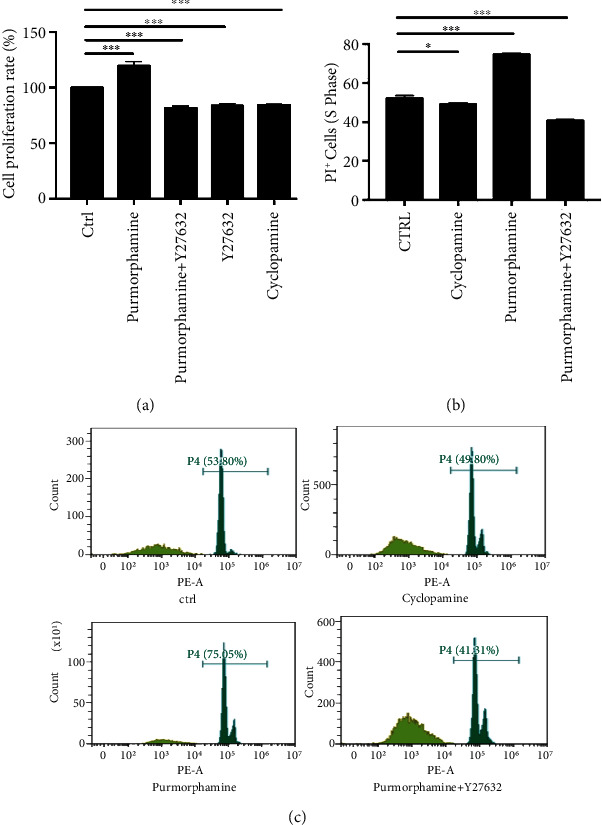
Shh signaling promotes the proliferation of RA-FLS *via* the Rho/ROCK pathway. (a) RA-FLS were treated with purmorphamine, purmorphamine combined with Y27632, Y27632, or cyclopamine for 48 h. The cell proliferation rate was measured with a CCK8 assay. (b, c) RA-FLS were treated with purmorphamine, purmorphamine combined with Y27632, or cyclopamine for 48 h, and the proportion of cells in the S phase was detected by flow cytometry. ^∗^*p* < 0.05, ^∗∗∗^*p* < 0.001.

To measure the proliferation activity of RA-FLS, we conducted a CCK8 assay. The results showed that the proliferation activity was significantly greater in the purmorphamine group (119.69 ± 3.71%) than in the control group (100 ± 0%) (*p* < 0.001) (Figure 4(a)). However, after the RA-FLS were treated with cyclopamine, Y27632, or a combination of purmorphamine and Y27632, the proliferation activity significantly decreased (81.55 ± 1.73%, 84.40 ± 0.70%, and 85.84 ± 0.81%, respectively, all *p* < 0.001) (Figure 4(a)).

### 3.5. Shh Signaling Regulates the Cell Cycle of RA-FLS *via* the Rho/ROCK Pathway

As shown in Figures 4(b) and 4(c), there was a significant increase in S-phase cells in the purmorphamine group compared to the control group (75.79% vs. 51.58%, *p* < 0.001), whereas the percentage in the cyclopamine group decreased (49.47% vs. 51.58%, *p* < 0.05). After the RA-FLS were treated with a combination of purmorphamine and Y27632, the proportion of cells in the S phase significantly decreased (41.05% vs. 51.58%, *p* < 0.001).

### 3.6. Shh Signaling Pathway Regulates Migration *via* Rho/ROCK Pathway in RA-FLS

**Figure 5:**
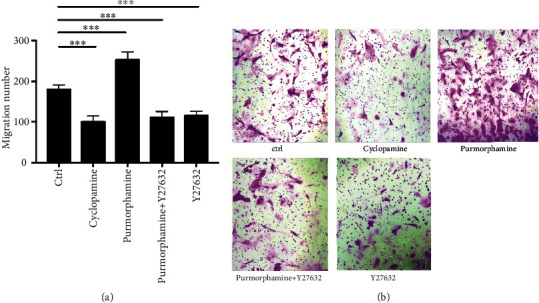
Shh signaling pathway regulates RA-FLS migration *via* Rho/ROCK pathway, tested with Transwell assays. RA-FLS were treated with purmorphamine, purmorphamine combined with Y27632, Y27632, or cyclopamine and the cell migration potency was evaluated with Transwell assays. (a) The photos of migrated RA-FLS were observed under an inverted microscope at 100x magnification. (b) The numbers of migrated RA-FLS were calculated under the microscope and displayed with a histogramat. ^∗∗∗^*p* < 0.001.

**Figure 6:**
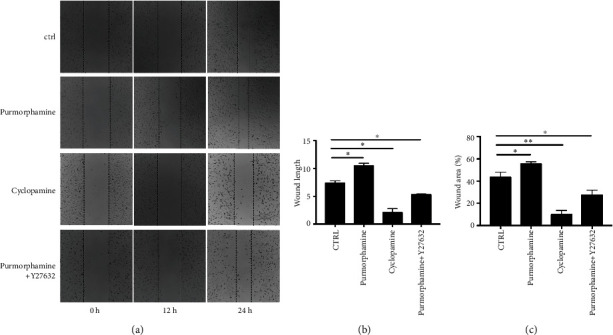
Shh signaling regulates RA-FLS migration *via* the Rho/ROCK pathway, revealed by wound healing assays. (a) In the Transwell assay, the numbers of migrated RA-FLS were higher in the treatment of purmorphamine; after RA-FLS treating with cyclopamine or purmorphamine combined with Y27632, the numbers of migrated cells were both decreased. (b) After 24 h, the wound healing duration of RA-FLS was increased as a result of purmorphamine treatment, while treated with cyclopamine or a combination of Purmorphamine and Y27632, it was decreased. (c) The wound healing area of RA-FLS in the treatment of purmorphamine was increased after 24 h, while it was decreased after treating with cyclopamine or a combination of purmorphamine for 24 h. ^∗^*p* < 0.05, ^∗∗^*p* < 0.01.

In the Transwell assay, we noted that the numbers of migrated (Figure 5) RA-FLS were significantly higher in the purmorphamine treatment group than in the control group (252.38 vs. 178.57, *p* < 0.001). Nonetheless, after the RA-FLS were treated with purmorphamine in the presence of Y27632, the number of migrated cells significantly decreased (109.52 vs. 178.57, *p* < 0.001). Similarly, the cell numbers for migration were also significantly reduced by inhibiting Smo with cyclopamine (100 vs. 178.57, *p* < 0.001). After 24 h, the wound healing duration and wound healing area (Figure 6) increased as a result of purmorphamine treatment (10.45 vs. 7.46 and 53.82% vs. 43.64%, both *p* < 0.05). In contrast, after the RA-FLS were treated with cyclopamine, the wound healing area (10.18% vs. 43.64%, *p* < 0.01) and the wound healing duration significantly decreased (2.09 vs. 7.46, *p* < 0.05). After the RA-FLS were treated with a combination of purmorphamine and Y27632, the wound healing duration and area also decreased significantly compared to the values in the control group (5.37 vs. 7.46 and 27.64% vs. 43.64%, both *p* < 0.05).

## 4. Discussion

RA-FLS display tumor-like features, including enhanced cell growth and migration ability *in vivo*, which contribute to the pathogenesis of cartilage and bone destruction [26]. Recent studies showed that RA-FLS can spread from destructive arthritis to other distant joints *via* the vasculature in mice with severe combined immunodeficiency, suggesting the characteristic clinical phenomenon of destructive arthritis spreading between joints might occur through the transmigration of activated FLS [27, 28]. Therefore, it is important to investigate how these tumor-like behaviors are enhanced in RA-FLS to identify new therapeutic targets.

Signaling pathways that play a crucial role during development may also be implicated in autoimmune diseases. In a previous study, we identified that Shh is activated in RA-FLS to promote both cell proliferation and migration. The expressions of Shh signaling molecules such as Ptch1, Smo, and Gli are also upregulated in RA patients compared to the levels in knee trauma patients [16, 17, 19]. Additionally, Shh signaling is transduced by Rho/ROCK in a noncanonical pathway [29]. In human endothelial cells, Hh isoforms induce the G-dependent activation of the monomeric G protein RhoA [30]. Furthermore, increasing evidence has shown that SMO initiates canonical and noncanonical responses to Hh stimulation *via* separate domains and G proteins play a central role in noncanonical signaling by linking Hh signaling to small Rho GTPase [31]. Therefore, the role of the Rho/ROCK pathway in RA-FLS stimulated by Shh signaling was important and being investigated in the present study. Our study suggests that Shh-Rho signaling may be a potential target to reduce cartilage damage in RA.

By using western blot assays, we further validated the impact of the Shh signaling pathway, which was stimulated by Smo, on Rho GTPase activation in RA-FLS. In accordance with previous studies [19], our results demonstrated that RhoA/ROCK signaling was activated by a Smo agonist whereas a specific inhibitor of Smo suppressed the activities of RhoA/ROCK signaling. Furthermore, our data showed that the Smo agonist, purmorphamine, induced the proliferation and migration of RA-FLS, and this effect was blocked by cyclopamine, an inhibitor of Smo. Moreover, we noted that Y27632 treatment also reversed the increased proliferation and migration induced by purmorphamine. The results indicate that Shh signaling plays an important role in the proliferation and migration of RA-FLS through the activation of Rho GTPase signaling. These findings of our study may provide a potential pharmaceutical target to inhibit the aggressive phenotype of RA-FLSs and restrain pathological synovial invasion in RA. Interestingly, previous studies provided evidence of the redistribution of the cell cycle induced by purmorphamine [16, 18]. Polizio et al. also found that multistage cell cycle arrests can be caused by the depletion of both ROCK1 and 2 [32]. In this study, we found that purmorphamine can increase the proportion of FLS cells in the S phase, which is the most important period for DNA replication in the cell cycle [33]. However, this tendency can be abolished by cyclopamine and Y27632, the inhibitors of Rho/ROCK. Nonetheless, whether Shh promotes the proliferation and migration of FLS via Rho/ROCK signaling through the cell cycle may warrant further research.

There were some limitations in this study. Firstly, the sample in this study was limited due to the synoviocytes were collected from patients undergoing knee arthroscopy and replacement. Secondly, the patients in our study were with long disease duration. Whether the Shh pathway plays the same role in patients with early RA remains to be further investigated.

In this study, we investigated the underlying mechanism(s) of the Shh signaling pathway for promoting proliferation and migration in RA-FLS. We identified crosstalk between Shh signaling and Rho/ROCK signaling, providing novel insights into the pathogenesis and potential therapeutic targets of RA.

## Data Availability

The datasets used and/or analyzed during the current study are available from the corresponding author on reasonable request.

## Abbreviations

RA: Rheumatoid arthritis
FLS: Fibroblast-like synoviocytes
Shh: Sonic hedgehog
Hh: Hedgehog.

## References

[1] Cush J. J. (2022). Rheumatoid arthritis: early diagnosis and treatment. *Rheumatic Diseases Clinics of North America*.

[2] Wu Y. Y., Li X. F., Wu S. (2022). Role of the S100 protein family in rheumatoid arthritis. *Arthritis Research & Therapy*.

[3] Nygaard G., Firestein G. S. (2020). Restoring synovial homeostasis in rheumatoid arthritis by targeting fibroblast-like synoviocytes. *Nature Reviews Rheumatology*.

[4] Liu Y., Pan Y. F., Xue Y. Q. (2018). uPAR promotes tumor-like biologic behaviors of fibroblast-like synoviocytes through PI3K/Akt signaling pathway in patients with rheumatoid arthritis. *Cellular & Molecular Immunology*.

[5] Kreider-Letterman G., Carr N. M., Garcia-Mata R. (2022). Fixing the GAP: the role of RhoGAPs in cancer. *European Journal of Cell Biology*.

[6] Mosaddeghzadeh N., Ahmadian M. R. (2021). The RHO family GTPases: mechanisms of regulation and signaling. *Cell*.

[7] Liang L. Q., Huang M. C., Qiu Q. (2013). Modulation of RhoA/Rho kinase on migration, invasion and proliferation of fibroblast like synoviocytes from patients with rheumatoid arthritis. *Zhonghua Yi Xue Za Zhi*.

[8] Zhang X., Mélik-Parsadaniantz S., Baudouin C., Réaux-Le Goazigo A., Moreau N. (2022). Shhedding new light on the role of hedgehog signaling in corneal wound healing. *International Journal of Molecular Sciences*.

[9] Fattahi S., Pilehchian Langroudi M., Akhavan-Niaki H. (2018). Hedgehog signaling pathway: epigenetic regulation and role in disease and cancer development. *Journal of Cellular Physiology*.

[10] Xiao Y. F., Sun Y., Liu W. (2021). HMGB1 promotes the release of sonic hedgehog from astrocytes. *Frontiers in Immunology*.

[11] Zhu S. L., Ye Y. M., Shi Y. M. (2020). Sonic hedgehog regulates proliferation, migration and invasion of synoviocytes in rheumatoid arthritis via JNK signaling. *Frontiers in Immunology*.

[12] Hashemitabar M., Heidari E. (2019). Redefining the signaling pathways from pluripotency to pancreas development: in vitro *β*-cell differentiation. *Journal of Cellular Physiology*.

[13] Zhong C. Q., Wang B. B. (2022). Regulation of cholesterol binding to the receptor patched 1 by its interactions with the ligand sonic hedgehog (Shh). *Frontiers in Molecular Biosciences*.

[14] Nguyen N. M., Cho J. (2022). Hedgehog pathway inhibitors as targeted cancer therapy and strategies to overcome drug resistance. *International Journal of Molecular Sciences*.

[15] Wei L. H., Arastoo M., Georgiou I., Manning D. R., Riobo-Del Galdo N. A. (2018). Activation of the Gi protein-RHOA axis by non-canonical Hedgehog signaling is independent of primary cilia. *PLoS One*.

[16] Zhu S. L., Huang J. L., Peng W. X. (2017). Inhibition of smoothened decreases proliferation of synoviocytes in rheumatoid arthritis. *Cellular & Molecular Immunology*.

[17] Wang M., Zhu S., Peng W. (2014). Sonic hedgehog signaling drives proliferation of synoviocytes in rheumatoid arthritis: a possible novel therapeutic target. *Journal of Immunology Research*.

[18] Liu F., Feng X. X., Zhu S. L. (2018). Sonic hedgehog signaling pathway mediates proliferation and migration of fibroblast-like synoviocytes in rheumatoid arthritis via MAPK/ERK signaling pathway. *Frontiers in Immunology*.

[19] Peng W. X., Zhu S. L., Zhang B. Y. (2017). Smoothened regulates migration of fibroblast-like synoviocytes in rheumatoid arthritis via activation of rho GTPase signaling. *Frontiers in Immunology*.

[20] Arnett F. C., Edworthy S. M., Bloch D. A. (1988). The American Rheumatism Association 1987 revised criteria for the classification of rheumatoid arthritis. *Arthritis and Rheumatism*.

[21] Su W., Fan H., Chen M. (2012). Induced CD4^+^ forkhead box protein-positive T cells inhibit mast cell function and established contact hypersensitivity through TGF-*β*1. *The Journal of Allergy and Clinical Immunology*.

[22] Pan W., Li W., Zhao J. (2019). lncRNA-PDPK2P promotes hepatocellular carcinoma progression through the PDK1/AKT/caspase 3 pathway. *Molecular Oncology*.

[23] Grada A., Otero-Vinas M., Prieto-Castrillo F., Obagi Z., Falanga V. (2017). Research techniques made simple: analysis of collective cell migration using the wound healing assay. *The Journal of Investigative Dermatology*.

[24] Sack U., Hirth A., Funke B. (2005). A novel model of fibroblast-mediated cartilage destruction. *Scandinavian Journal of Immunology*.

[25] Mo B. Y., Guo X. H., Yang M. R. (2018). Long non-coding RNA GAPLINC promotes tumor-like biologic behaviors of fibroblast-like synoviocytes as microRNA sponging in rheumatoid arthritis patients. *Frontiers in Immunology*.

[26] Taghadosi M., Adib M., Jamshidi A., Mahmoudi M., Farhadi E. (2021). The p53 status in rheumatoid arthritis with focus on fibroblast-like synoviocytes. *Immunologic Research*.

[27] Luo Y., Wu W., Gu J. (2019). Human gingival tissue-derived MSC suppress osteoclastogenesis and bone erosion via CD39-adenosine signal pathway in autoimmune arthritis. *eBioMedicine*.

[28] Chen W., Wang J., Xu Z. (2018). Apremilast ameliorates experimental arthritis via suppression of Th1 and Th17 cells and enhancement of CD4(+)Foxp3(+) regulatory T cells differentiation. *Frontiers in Immunology*.

[29] Renault M. A., Roncalli J., Tongers J. (2010). Sonic hedgehog induces angiogenesis via Rho kinase-dependent signaling in endothelial cells. *Journal of Molecular and Cellular Cardiology*.

[30] Iriana S., Asha K., Repak M., Sharma-Walia N. (2021). Hedgehog signaling: implications in cancers and viral infections. *International Journal of Molecular Sciences*.

[31] Sigafoos A. N., Paradise B. D., Fernandez-Zapico M. E. (2021). Hedgehog/GLI signaling pathway: transduction, regulation, and implications for disease. *Cancers (Basel)*.

[32] Polizio A. H., Chinchilla P., Chen X., Kim S., Manning D. R., Riobo N. A. (2011). Heterotrimeric G_i_ proteins link Hedgehog signaling to activation of Rho small GTPases to promote fibroblast migration∗. *The Journal of Biological Chemistry*.

[33] Bertoli C., Skotheim J. M., de Bruin R. A. (2013). Control of cell cycle transcription during G1 and S phases. *Nature Reviews. Molecular Cell Biology*.

